# Gastrotomy for the Removal of an Ovarian Tumor; Error of Diagnosis; Peculiar Condition of the Abdominal Parietes and Contents; Patient Recovered

**Published:** 1855-01

**Authors:** Henry H. Smith

**Affiliations:** Consulting Surgeon and Lecturer on Clinical Surgery in the Philadelphia Hospital; Philadelphia


### THE
### MEDICAL EXAMINER.

NEW SERIES.—NO. CXXI.—JANUARY, 1855.

### ORIGINAL COMMUNICATIONS.

*Gastrotomy for the Removal of an Ovarian Tumor; error of diagnosis; peculiar condition of the abdominal parietes and contents; patient recovered.* By Henry H. Smith, M. D., Consulting Surgeon and Lecturer on Clinical Surgery in the Philadelphia Hospital.

The Philadelphia Hospital, being that portion of the Philadelphia Alms-House which is occupied by the sick poor, is an institution which contains upwards of 800 beds, about 160 of which are occupied by surgical cases. With so large a number of patients, and with a lecture room capable of accommodating 1000 students, it presents us with one of the best clinical schools in the United States. After being for nine years closed to the medical classes of Philadelphia, it was again opened for their instruction in September, 1854.

Soon after entering upon my duties as one of the Consulting Surgeons, I was informed by the Chief Resident Physician, that among other cases requiring operative treatment, was one of ovarian tumor of some standing, in which the patient was so anxious for an operation that she had recently eloped from the institution in order to gain her wishes; he having refused to operate only because she was not in a proper condition. This patient being subsequently presented to my notice, I made an examination of her abdomen, but not being satisfied as to the character of her complaint, deferred the expression of an opinion until I could learn more of her history. This, together with the notes of her subsequent condition, being kindly obtained for me by Dr. G. B. Smith, of Tennessee, one of the Residents in charge of the clinical wards, are now presented in a condensed form. To Dr. G. B. Smith, and also to his colleague Dr. T. Braxton, of Va., I feel indebted for the interest displayed in the treatment of the case, as well as for the untiring assiduity with which they attended to the patient for several days after the operation.

*Case of Abdominal Tumor. General History.*—Maria W., aged 23 years, of sanguine temperament and considerable embonpoint, weighing about 155 pounds, was born in the interior of Pennsylvania. At the early age of 14 years she ran away from home, and lived with her seducer until she bore him two children. Being soon after deserted by him, "she lived upon the town," and on one occasion was said to have submitted to the embraces of thirteen men in a few hours. She has had four children, only one of whom is alive; had one child delivered by the operation of embryulcia, and has repeatedly suffered from syphilis, for which she was treated in the hospital. On one occasion she was found to have nine distinct chancres on the neck and mouth of the uterus. It was soon after this visit that she again became an inmate of the hospital, on account of severe abdominal pain and swelling, for which she was repeatedly cupped, blistered, &c., the marks of which are yet very apparent. This visit she thinks was about eight months since, but she has no distinct recollection of the period "when the lump first appeared; though different physicians in the city had treated her for the lump, and she had spent all her money in trying to get rid of it" before she came to the hospital. Such was her history up to the period of my visit, except that "her doctors always told her she had a lump like her sister." This sister had a tumor in the abdomen, of which she died in about 12 months, and on being examined after death, the tumor was found to be filled with water, and hence her anxiety to be operated on promptly.

After receiving this account I made a most careful examination, and found the following condition of things :

*Present Condition.*—General health fair, strength good, countenance intelligent, but expressive of temper which is excessive; limbs round and full, but without œdema; pulse natural; abdomen very tumid, especially on the left side of the umbilical, as well as the left hypochondriac and iliac regions, where a globular tumor of the full size of an adult head is perceptible: the skin over the tumor is dark colored and marked by cups; no wrinkle of skin from former pregnancies, owing to the general abdominal distension, which is equal to that of six months' pregnancy; pressure on the left iliac region causes pain; indistinct sense of fluctuation in the tumor and also in the abdomen. Umbilicus quite prominent, and resembling the appearance of a small irreducible umbilical hernia.

The tumor on the left side of the abdomen is irregular on the surface though not lobulated, is *flat on percussion* when the patient lies on her back, but less dull when she lies on her right side. When placed on her right side the tumor falls considerably to the right of the linea alba, and is also moveable in its pelvic connections. The solidity and mobility of the tumor was therefore undoubted.

As Maria was suffering much from hemorrhoids, no examination was made per rectum, but that per vaginam showed a marked prolapsus uteri, with great tumefaction of the neck and eversion of the os. There was also considerable fulness of the anterior and left wall of the vagina. The bladder exhibited considerable irritation, and required the frequent use of the catheter, the difficulty in micturition being apparently due to a tumor within the pelvis which depressed the womb and thus acted on the bladder.

In this examination several of the Resident Physicians and one or two others participated. Knowing the difficulties attendant on the diagnosis of ovarian tumors, I made in all four very careful examinations of the patient, and became satisfied that the tumor was ovarian, that it was comparatively free from adhesions, that it was the cause of her vesical and rectal distress, and that the attempt to remove it was justifiable under the circumstances. In this opinion my colleagues Drs. D. Hayes Agnew and A. B. Campbell fully coincided. After due preparation and after a full explanation to the patient of the dangers of the operation, and the probability of our not being able to relieve her, no change was made in her anxiety to submit to it, and she was accordingly prepared therefore by moderate purging with pills of inspissated ox gall; the bladder evacuated; the pelvis surrounded by a large diaper and an anæsthetic (ether 5 parts, chloroform 1) administered. Whilst these arrangements were being effected in the ward, I explained the character of the complaint and the operation about to be performed, to the large class in attendance, stating that the greatest difficulty in all such cases was the inability of the surgeon to make a positive diagnosis before opening the abdomen.

*Operation.*—The patient being now placed on the operating table, in a perfect state of anæsthesia, the attention of the class was called to the tumid condition of the abdomen generally; to the apparent existence of a slight umbilical hernia, and to the size and position of the ovarian tumor. Whilst all her muscles were thus relaxed by the ether, I, however, thought that the tumor was not so prominent as it had appeared to me on a former occasion, but as this was deemed by all near the table to be due to the absence of the compression made upon it by the contraction of the abdominal muscles, and as the tumor was yet perfectly distinct and moveable, no importance was attached to the observation. Accordingly, I proceeded to operate, assisted by Drs. Agnew and Campbell, and Drs. G. B. Smith and Braxton, and others of the House Physicians. Commencing a little to the left of the umbilicus, I divided the integuments to the extent of eight inches terminating within an inch of the pubis. On carefully dividing the tendon of the external oblique muscle, my finger touched the peritoneum, the linea alba being deficient, and the recti muscles separated at the umbilicus to the extent of one inch and a half by the great abdominal distension. On carefully incising the peritoneum, the omentum majus was found fully spread out over the intestines, thickened to nearly a half inch and filled by a lump-like and fatty deposit. It was also adherent to all the surrounding parts. After destroying some of the adhesions, the hand was passed into the left iliac region to feel the base of the tumor, when the ovary was discovered to be sound, though the uterus was somewhat, though not considerably enlarged. As the tumor yet apparently existed beneath the abdominal parietes, the hand was carried more towards the left hypochondrium, and as the patient at this moment began to move, about fifteen feet of intestines protruded at the wound. These were very much glued together by adhesions due evidently to old peritonitis, and the tumor had doubtless been caused by the adhesion of a large intestinal convolution beneath a mass of indurated omentum ; for on destroying the adhesions I destroyed also the globular form, and left nothing but an omental tumor. The wound was therefore closed by several points of the twisted suture, covered by adhesive strips and supported by a compress and bandage. On being placed in bed her pulse was 120, and as the effects of the anæsthetic passed off, she became restless, to relieve which she took a half grain of sulphate of morphia. Three hours subsequently her pulse was 78, her urine was drawn off, and she slept. From this time her treatment, which was carefully noted by Drs. Smith and Braxton, consisted in free doses of anodyne, of which she generally took a grain and a half of sulphate of morphia per diem ; in the constant use of the catheter to prevent distension of the bladder, and in the close observance of an equable temperature, light diet, &c. On the 6th day the sutures were removed, and nearly the entire wound found to be healed by the first intention. On the 8th day her bowels were evacuated by an injection, being the first time since the operation, and on the 21st day she was able to present herself to the class, having never had a serious symptom. She is now able to move about freely, but yet suffers from hemorrhoids. Owing to the condition of her recti muscles, she wears a broad belt over her abdomen to prevent any tendency to hernial protrusion, the umbilicus being yet as patulous as it was prior to the operation.

*Remarks.*—The propriety of even attempting the relief of ovarian tumors by the operation of ovariotomy is a surgical question that must be regarded as yet unsettled, the very conservative spirits in the profession looking on it with great repugnance, and the more progressive urging its performance, as the least dangerous method of accomplishing a cure. When undertaken with ordinary skill, the operative proceeding does not present any great difficulty, and the results of the case that have been reported certainly show that the risks of peritonitis are not so great in abdominal tumors, especially when adherent as they were formerly supposed to be. But the difficulties of diagnosis, and the numerous instances in which the operation has failed in the accomplishment of its object, multiply daily, and must be regarded as serious obstacles to the establishment of ovariotomy as a settled point of practice for the relief of these tumors. Unless surgeons, when tempted to operate, place their cases upon record without any reference to the success which attends their efforts, no true progress can be made towards the settlement of this question.

The failures which are due to errors of diagnosis are not rare. Lizars of Edinburg, in 1825, reported a case similar to the preceding one, the recti muscles being separated by the distension, the abdomen laid open, and yet no tumor found, owing, as he remarks, "to the great obesity of the patient and the distended fulness of the intestines.* In a second case, the tumor could not be removed in consequence of the enlarged and adherent condition of the omentum. Dr. Bright† also mentions a case in which after the abdomen was opened no tumor was found. Dieffenbach‡ attempted the removal of an ovarian tumor, but after laying open the abdomen found a tumor connected to the vertebra, which contained vessels that pulsated with great force, and on being punctured gave rise to profuse hemorrhage and symptoms of intestinal strangulation, though the patient recovered. Dr. Dohloff§ opened the abdominal cavity for the removal of a tumor, but after searching for it found none. Mr. South|| states that Jeaffreson, in his tables, reports 23 cases out of 74 in which the diagnosis was not sufficiently accurate to enable the surgeon to foresee the impracticability of carrying out his intentions. In 14 of these 23 there were adhesions to such an extent as to preclude removal, in 3 no tumor was found, and in 6 the tumor proved

* Lizar's Observ. Extirpation of Diseased Ovaria, p. 6 and 7, Ed. 1825.

† Bright on Abdominal Tumors. Guy's Hosp. Reports, vol. 3, p. 257, 1838.

‡ British and Foreign Med. Review, vol. 16, p. 400, 1843.

§ Brit. and Foreign Med. Rev. vol. 16, p. 401, from Rust's Magazine, 1838.

|| Chelius, Philada. edit., vol. 3, p. 213.

to be other than ovarian. Dr. Washington L. Atlee* refers to 222 cases of ovariotomy, in 6 of which there was no tumor, or 1 in every 37 cases.

Although these references show that the errors of diagnosis in abdominal or supposed ovarian tumors are not rare, there is, I fear, reason to think, that if all the errors had been published, the number would have been much augmented, two cases having come to my knowledge in which there was no tumor, though the operator was very explicit in stating the infallible signs of its existence prior to the operation. Where truth is stated and facts multiplied by publication, a correct result must be obtained in any question, and it is to be hoped that no surgeon who feels justified in performing ovariotomy will hesitate in placing his operation on record for the benefit of the profession, no matter what may be the result. He who is not conscious of having made an error of diagnosis, must either have been deficient in opportunities of investigating and treating disease, or be wrapped in an impenetrable mantle of self conceit.

*Philadelphia, November* 1854.

* Table of all the known operations of Ovariotomy, p. 28. Phil., 1851.

