# Erratum

**Published:** 1855-02

**Authors:** 


ERRATUM.—In Dr. H. H. Smith's article on "Gastrotomy," in our last Number, the word *lump-like*, on page 4, should read *lymph-like*.

